# Numerical and Interpretive Comparability of Optical and Mechanical Coagulation Analyzers: A Zlog-Supported Method Comparison Study

**DOI:** 10.3390/diagnostics16162511

**Published:** 2026-08-09

**Authors:** Xinjian Cai, Wei Yang, Qiuxia Lu, Yiteng Lin

**Affiliations:** Department of Clinical Laboratory Medicine, National Cancer Center/National Clinical Research Center for Cancer/Cancer Hospital & Shenzhen Hospital, Chinese Academy of Medical Sciences and Peking Union Medical College, Shenzhen 518116, China; luqiuxia@cicams-sz.org.cn (Q.L.); linyiteng@cicams-sz.org.cn (Y.L.)

**Keywords:** *zlog*, method comparison, coagulation testing, reference interval, harmonization

## Abstract

**Background/Objectives:** In laboratories using coagulation analyzers with different clot-detection principles and platform-specific reference intervals (RIs), numerical agreement does not necessarily ensure concordant clinical interpretation. We evaluated how analytical agreement and RI-based interpretation diverge between mechanical and optical coagulation analyzers. **Methods:** Paired duplicate measurements were performed on the STA R Max (Stago) and ACL TOP 750 LAS (Werfen) for prothrombin time (PT), activated partial thromboplastin time (APTT), thrombin time (TT), fibrinogen (FIB), international normalized ratio (INR), and antithrombin (AT). Numerical agreement was assessed by Passing–Bablok regression and log-ratio Bland–Altman analysis against predefined total allowable error (TEa) limits. Each result was standardized using its platform-specific RI, and the between-platform *zlog* difference (Δ*zlog*) described differences in RI-relative position. **Results:** Stago yielded higher values than Werfen for PT, APTT, TT, and FIB, with mean biases of +15.6% to +20.4%, whereas INR and AT showed close agreement (−1.1% and +1.6%). Interpretive concordance among the primary assays ranged from 61.9% for APTT to 89.2% for FIB. The analytical-by-interpretive matrix identified specimens that were within TEa but classified differently and specimens that were beyond TEa but retained the same RI classification. Median Δ*zlog* values were −0.43 for PT, +0.09 for APTT, −0.66 for TT, and +0.77 for FIB. **Conclusions:** Numerical and interpretive comparability are distinct dimensions. Combining the analytical-by-interpretive matrix with Δ*zlog* can reveal clinically relevant divergence not apparent from bias-versus-TEa assessment alone. In this purposively selected dataset, divergence arose mainly from RI offset for TT and analytical dispersion for APTT; the sample-specific discordance rates are not population prevalence estimates.

## 1. Introduction

Prothrombin time (PT), activated partial thromboplastin time (APTT), fibrinogen (FIB) and thrombin time (TT) are core coagulation assays that guide decisions on bleeding, thrombosis and anticoagulant therapy. Many laboratories run more than one analyzer, often based on different clot-detection principles—optical (photometric) and mechanical (viscosity-based) [1,2,3]—and the comparability of results for the same analyte across analyzers must be verified in accordance with quality standards such as CLSI EP09 and WS/T 407 [4,5]. Yet a numerically similar result can fall on opposite sides of platform-specific reference limits. Such platform-dependent flagging may affect repeat testing, further evaluation, or longitudinal interpretation when patients are tested across platforms.

PT, APTT, and FIB are not harmonized across manufacturers; results are reagent- and instrument-dependent, and even different evaluation modes on one analyzer can diverge, so system-specific reference intervals are recommended, and patient-level interpretation can differ between laboratories [3,6,7]. Therefore, each platform legitimately carries its own reference interval, and two analyzers may disagree on a number because they are anchored to different reference populations rather than because either is in error. Conventional comparability assessment by Passing–Bablok regression [8] and Bland–Altman analysis [9] judged against an allowable total error (TEa) operates on raw values and answers whether two instruments produce the same number; it does not establish whether a difference changes the laboratory interpretation when each platform has its own reference interval.

The *zlog* transformation rescales a result relative to its own reference limits onto a dimensionless scale, mapping the limits to ±1.96 [10]; it has been used for standardized reporting, plausibility checking of reference intervals, exploratory analysis, and machine learning [11,12,13,14]. We use *zlog* here to express, as a continuous between-platform difference (Δ*zlog*), how far each platform places a specimen from its own reference limits. Because categorizing a *zlog* value at ±1.96 is identical to categorizing the raw value at the same limits, the contribution is not a new classifier but the explicit separation of numerical from interpretive comparability: a 2 × 2 analytical-by-interpretive matrix that shows when an analytical difference does and does not change the laboratory flag, with Δ*zlog* as a continuous descriptor of standardized position. Because the *zlog* scaling factor is inversely proportional to reference-interval width, we interpret the sign and pattern of Δ*zlog* rather than treating its absolute value as directly comparable across assays. We therefore separate numerical comparability from reference-interval-anchored interpretation, aiming to demonstrate that the interpretive consequence of a given bias varies by assay; the design is not intended to estimate the population frequency of discordance.

## 2. Materials and Methods

### 2.1. Design and Analyzers

This single-center, retrospective method-comparison study used de-identified residual citrated plasma (3.2% sodium citrate, 9:1) from routine testing, following the general principles of CLSI EP09c and WS/T 407 [4,5]. The study was reviewed by the Chinese Academy of Medical Sciences and Peking Union Medical College Shenzhen Hospital Ethics Committee (acceptance No. IIT2026-18-1) and granted a waiver of specific informed consent, because it used only residual specimens remaining after routine clinical testing, for which the patients had already provided general informed consent for research use of residual samples; the study therefore posed no additional risk to patients. Two analyzers were compared: the mechanical, viscosity-based STA R Max (Diagnostica Stago, Paris, France) and the optical, photometric ACL TOP 750 LAS (Instrumentation Laboratory, Werfen Group, Bedford, MA, USA), each with the manufacturer’s reagents and calibrators used according to the manufacturers’ instructions. On the STA R Max, PT used STA-Neoplastine CI Plus (rabbit-brain thromboplastin, ISI 1.36; lot 274436), APTT used STA-PTT A (silica activator; lot 273658), TT used STA-Thrombin (lot 273549), fibrinogen used STA-Fibrinogen (Clauss method; lot 273508), antithrombin used the chromogenic STA-Stachrom AT (lot 272513), and D-dimer used STA-Liatest D-Di (lot 273351); calibration used STA Unicalibrator (lot 271285) and quality control used STA Coag Control N + P (lot 272504). On the ACL TOP 750 LAS, PT used HemosIL RecombiPlasTin 2G (recombinant human tissue factor, ISI 1.01; lot N0552686), APTT used HemosIL SynthASil (colloidal-silica activator; lot N0552678), TT used HemosIL Thrombin Time (lot N0855365), fibrinogen used HemosIL Fibrinogen-C XL (Clauss method; lot N0553485), antithrombin used the chromogenic HemosIL Liquid Antithrombin (lot N0654323), and D-dimer used HemosIL D-Dimer HS 500 (lot B37681); calibration used HemosIL Calibration Plasma (lot E0743942) and quality control used HemosIL Normal, Low Abnormal and High Abnormal Assayed Controls, Low Fibrinogen Control and D-Dimer HS 500 Controls. INR was derived from PT using each reagent’s ISI, and both APTT reagents are silica-activated.

### 2.2. Samples and Anticoagulant Status

Residual citrated-plasma specimens from routine inpatient and outpatient testing performed between 1 May and 20 June 2026 were selected purposively to ensure representation across the analytical and interpretive range of each assay, including results within the RI and abnormal results near or beyond clinical decision limits. This range-enriched strategy increased the information available for method comparison and boundary-discordance analysis, but it did not reproduce the distribution of results in routine practice. Accordingly, all concordance and discordance rates describe this study sample and are not prevalence estimates for an unselected clinical population. Documented exposure to anticoagulant and antiplatelet agents was assessed for each specimen from the electronic medical record and laboratory order information. Documented therapy was present in three specimens: warfarin in one, rivaroxaban in one, and aspirin in one. No such therapy was documented for the remaining specimens; however, undocumented medication use could not be fully excluded. Because oral anticoagulant exposure may affect routine clotting assays, with reagent-dependent effects particularly relevant for direct oral anticoagulants such as rivaroxaban [15], sensitivity analyses excluding the warfarin and rivaroxaban specimens were performed for PT and APTT. The aspirin specimen was retained, as aspirin affects platelet function but is not expected to directly prolong routine plasma clotting times.

One AT specimen (ID 3-022) was excluded from the AT analysis because one of the duplicate Stago measurements was inconsistent with the paired replicate results (114% and 220%) and exceeded the expected duplicate imprecision observed in this study. The 220% value was considered an isolated analytical outlier in the absence of concordant results from the paired measurements. Therefore, the corresponding AT pair was excluded before agreement analysis, leaving 54 evaluable AT pairs. D-dimer was not included among the primary analytes because only 25 paired results were available; it was therefore reported as an exploratory comparison only. PT activity (Quick %) was not analyzed separately because it is derived from the PT system and provides information overlapping with PT seconds and INR.

TT was analyzed in two predefined datasets. The primary dataset comprised 59 specimens selected to span the interpretive range from normal to prolonged without conditioning selection on the flag from either platform. This dataset was used for the primary concordance and discordance analyses; however, its range-enriched purposive design means that the resulting rates describe this study sample and are not prevalence estimates. A second dataset of 30 specimens was enriched for prolonged results, defined as a Werfen TT above the upper RI limit (>16.6 s). These specimens were selected on the Werfen value before the corresponding Stago results were reviewed and were used only for sensitivity and mechanism analyses. Because this one-platform enrichment would increase the observed discordance rate, the combined dataset (*n* = 89) was not used for the primary rate estimate.

### 2.3. Reference Intervals and Zlog

Manufacturer-specified RIs were used for each analyzer (Table 1) after local verification, conducted separately from the paired residual-specimen comparison, in apparently healthy adults from the local population according to CLSI EP28-A3C and WS/T 402-2024 [16,17]. For each analyte-specific interval or sex-/age-specific subgroup where applicable, at least 20 reference individuals were tested. An RI was accepted if no more than 2 of 20 results fell outside the proposed limits. If 3 or 4 results were outside, verification was repeated using an independent set of 20 reference individuals; de novo establishment was required only if repeat verification also failed. All RIs used in this study passed verification, and none required de novo re-establishment. The individual-level demographic records and exact out-of-range counts for these verification cohorts were not retained with the paired analytical dataset, which limits more granular retrospective reporting of the verification population.

Local transference verification was performed using reference individuals from the local population. The TT reference interval was higher on the Stago analyzer than on the Werfen analyzer (14–21 s vs. 10.3–16.6 s), consistent with differences in the reagent systems and clot-detection principles. Because the two intervals overlapped only between 14.0 and 16.6 s, TT results between 16.6 and 21.0 s would be classified as prolonged by Werfen but within the reference interval by Stago. This interval configuration created a platform-specific classification zone relevant to the TT interpretive discordance analyzed below.

The *zlog* transformation was selected because it places results from analyzers with different verified RIs on a common, continuous RI-relative scale. On the logarithmic scale, it accounts for both the midpoint and width of each platform’s RI and maps the lower and upper limits to −1.96 and +1.96, respectively. This makes the direction and dispersion of between-platform differences interpretable in relation to each platform’s own limits without imposing a common RI. For each result *x*, the *zlog* value was calculated as follows [10]:

$$zlog\left(x\right)=\frac{\left[\ln x-\frac{\ln LL+\ln UL}{2}\right]\times 3.92}{\ln UL-\ln LL}$$

where *LL* and *UL* are the lower and upper reference limits, so that the limits map to ±1.96. Each analyzer used its own interval, and Δ*zlog* = *zlog*_Stago_ − *zlog*_Werfen_.

Because each result was standardized using the limits of its own analyzer, Δ*zlog* describes the between-platform difference in standardized RI-relative position rather than a difference against a single common reference scale. Categorization at ±1.96 is identical to classification against the original RI; *zlog* is therefore an analytical comparison tool, not a new clinical decision rule. Since the scaling factor, 3.92/(ln *UL* − ln *LL*), is inversely related to RI width, Δ*zlog* magnitudes are not directly comparable across assays. We interpreted Δ*zlog* by its direction and distribution: a directional shift supports systematic RI offset, whereas a broad distribution around zero supports heterogeneous analytical dispersion.

### 2.4. Statistical Analysis

Each specimen was measured in duplicate on each analyzer, and the mean was used for analysis. Duplicate results estimated within-run repeatability in this dataset following the general principles of CLSI EP15, with replicate CVs of approximately 1–3% [18]. Passing–Bablok regression [8], implemented with the R package mcr [19], was selected because it is nonparametric, does not assume a specific error distribution, and accommodates measurement error in both methods. Bland–Altman analysis [9] was used to estimate bias and 95% limits of agreement directly; log ratios expressed proportional differences on an interpretable percentage scale. Passing–Bablok results are reported as slope and intercept with 95% confidence intervals, and Bland–Altman results as percentage bias and 95% limits of agreement. For APTT and TT, limited effective range, clustered values or ties, and substantial scatter made the Passing–Bablok estimates unstable; these estimates were therefore interpreted cautiously and together with the Bland–Altman and graphical results. Lin’s concordance correlation coefficient was not selected as a primary metric because it is strongly influenced by the sampled range and distribution; in this purposively range-enriched dataset, a single range-sensitive coefficient could obscure local bias and RI-based discordance. The combined regression, bias/limits, TEa, categorical concordance, and Δ*zlog* analyses were chosen to address the study’s numerical and interpretive objectives directly. The TEa limits used in this study were the acceptance limits of the Chinese national external quality assessment scheme administered by the National Center for Clinical Laboratories. For context, desirable TEa estimates derived from biological variation are lower, approximately 4% for PT, 7% for APTT, and 14% for FIB. These estimates were based on within- and between-subject biological variation data from the EFLM Biological Variation Database [20] and the desirable analytical-performance-specification model [21]; biological variation in coagulation assays has also been evaluated in dedicated studies [22].

In the analytical-by-interpretive framework, the choice of TEa affects only the analytical axis, namely the classification of specimens as within or beyond the allowable error. Interpretive concordance and Δ*zlog* are determined by each result’s position relative to the corresponding analyzer-specific reference interval and are therefore independent of the TEa threshold. For the analytical axis of the 2 × 2 matrix, each specimen was classified as within or beyond TEa according to whether the absolute percentage difference between platforms, ∣Stago − Werfen∣/[(Stago + Werfen)/2], was < the assay-specific TEa. This specimen-level classification defined the row split of the matrix and the “within TEa %” column in Table 2.

Interpretive concordance was assessed by comparing analyzer-specific reference-interval classifications. For PT, APTT, and TT, results were classified as normal or prolonged; for FIB and AT, results were classified as low, within the reference interval, or high. This classification is equivalent to applying *zlog* thresholds of ±1.96 after transformation using each analyzer’s own reference interval. Concordance and discordance proportions were summarized with Wilson 95% CIs. Cohen’s kappa [23] was calculated as an additional measure of categorical agreement, using the unweighted statistic for two-category assays and quadratic-weighted kappa for three-category assays [24]; kappa values were interpreted according to Landis and Koch [25]. Because the purposive sampling strategy did not preserve the prevalence of abnormal results, kappa was treated as a secondary descriptor and was reported with bootstrap 95% CIs. Δ*zlog* was reported as a continuous descriptor of between-platform differences in standardized reference-interval position, with bootstrap 95% CIs, and was summarized together with the analytical-by-interpretive 2 × 2 matrix. Bootstrap 95% CIs were calculated using the percentile method with 2000 resamples and a fixed random seed for reproducibility. Analyses were performed in Python version 3.14.5 (Python Software Foundation; NumPy 2.4.6, SciPy 1.17.1, and Matplotlib 3.10.9) and R version 4.5.1 (R Foundation for Statistical Computing, Vienna, Austria; R packages mcr 1.3.3.1 and ggplot2 4.0.0).

## 3. Results

**Figure 1:**
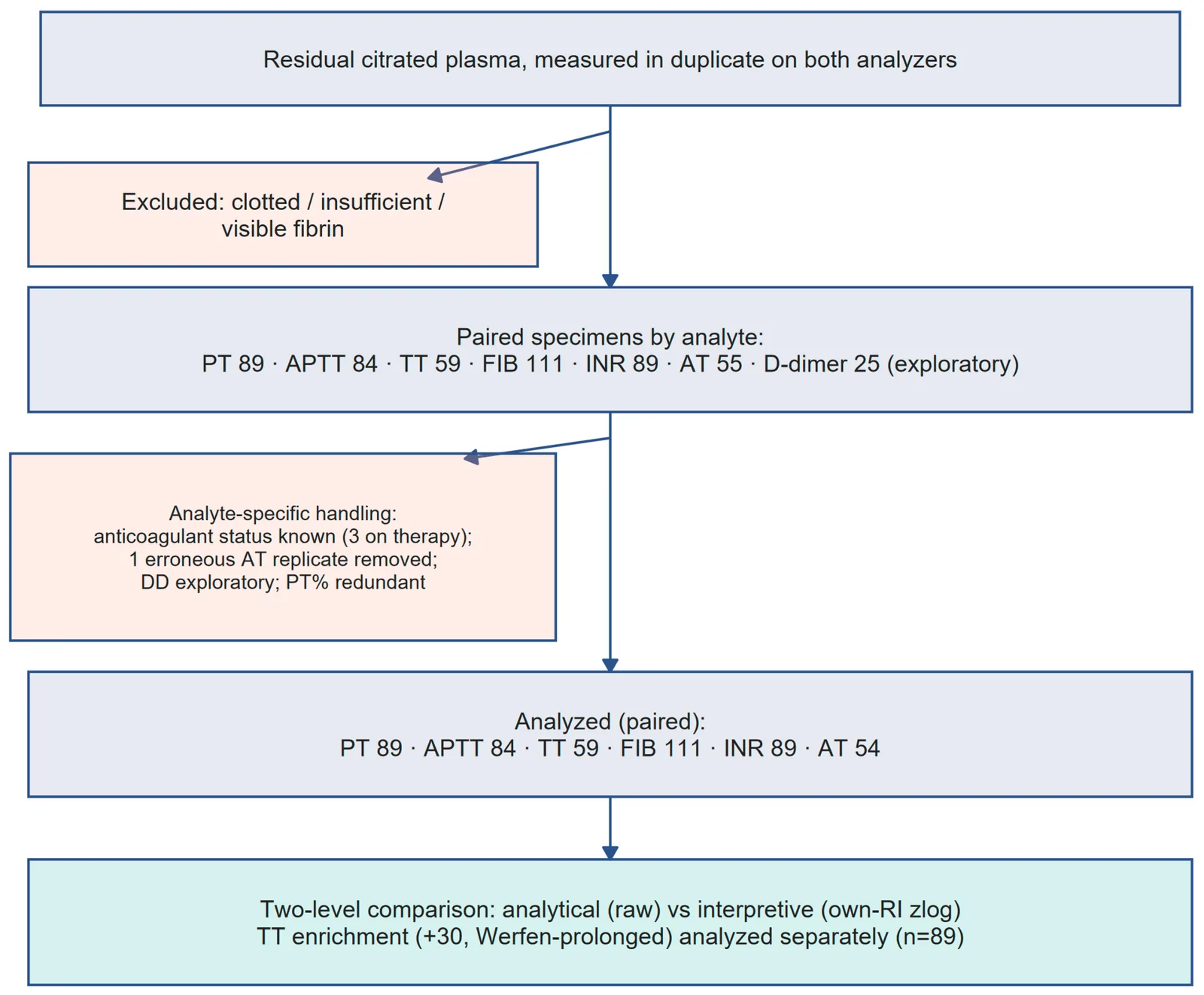
Specimen enrollment and analyte-specific handling.

Paired duplicate measurements were available for 89 PT, 84 APTT, 59 TT, 111 FIB, 89 INR, and 54 AT specimen pairs (Figure 1). The TT primary analysis was based on the non-enriched set of 59 specimens, with 30 additional Werfen-prolonged specimens analyzed separately.

### 3.1. Numerical Comparability

**Figure 2:**
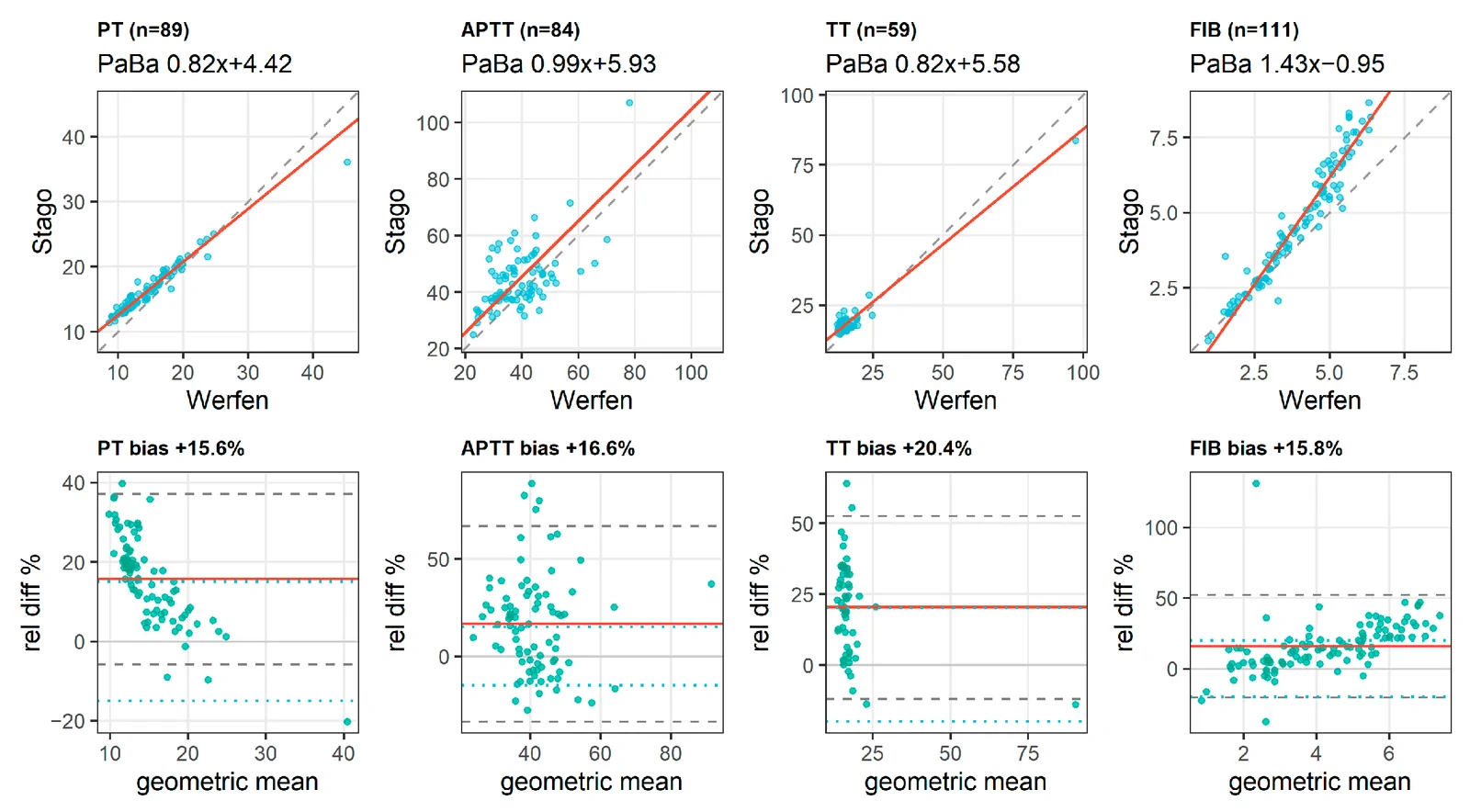
Analytical comparability of the primary assays (columns: PT, APTT, TT, and FIB). Top panels, Passing–Bablok regression (solid colored line) with the line of identity (diagonal dashed line). Bottom panels, log-ratio Bland–Altman plots with mean bias (solid line), 95% limits of agreement (dashed lines), and positive and negative TEa thresholds (dotted lines). These line types provide visual cues for systematic bias, dispersion, and clinically relevant analytical thresholds. TT uses the non-enriched primary set (*n* = 59).

Stago yielded systematically higher results than Werfen for the three clotting-time assays, with Bland–Altman biases of +15.6% for PT, +16.6% for APTT, and +20.4% for TT, and also for FIB (+15.8%) (Table 2; Figure 2). In contrast, between-platform biases were small for INR (−1.1%) and AT (+1.6%). FIB showed evidence of proportional bias, with a Passing–Bablok slope of 1.43. Passing–Bablok estimates for APTT and TT were unstable because of the limited effective measurement range for APTT and substantial scatter for TT; therefore, analytical agreement for these assays was interpreted primarily from the log-ratio Bland–Altman bias and limits of agreement, with residual plots shown in Figure S2. At the upper reference limit, the predicted bias exceeded the TEa limit for PT (+17.0% vs. ±15%), was close to the TEa limit for APTT (+15.1% vs. ±15%), and remained within TEa for TT (+15.9% vs. ±20%) and FIB (+18.8% vs. ±20%). Exclusion of the warfarin and rivaroxaban specimens did not materially change the PT or APTT results: PT, *n* = 87, bias +15.7% and interpretive concordance 88.5%; APTT, *n* = 82, bias +16.6% and interpretive concordance 61.0%.

### 3.2. Two Mechanisms of Interpretive Divergence

**Figure 3:**
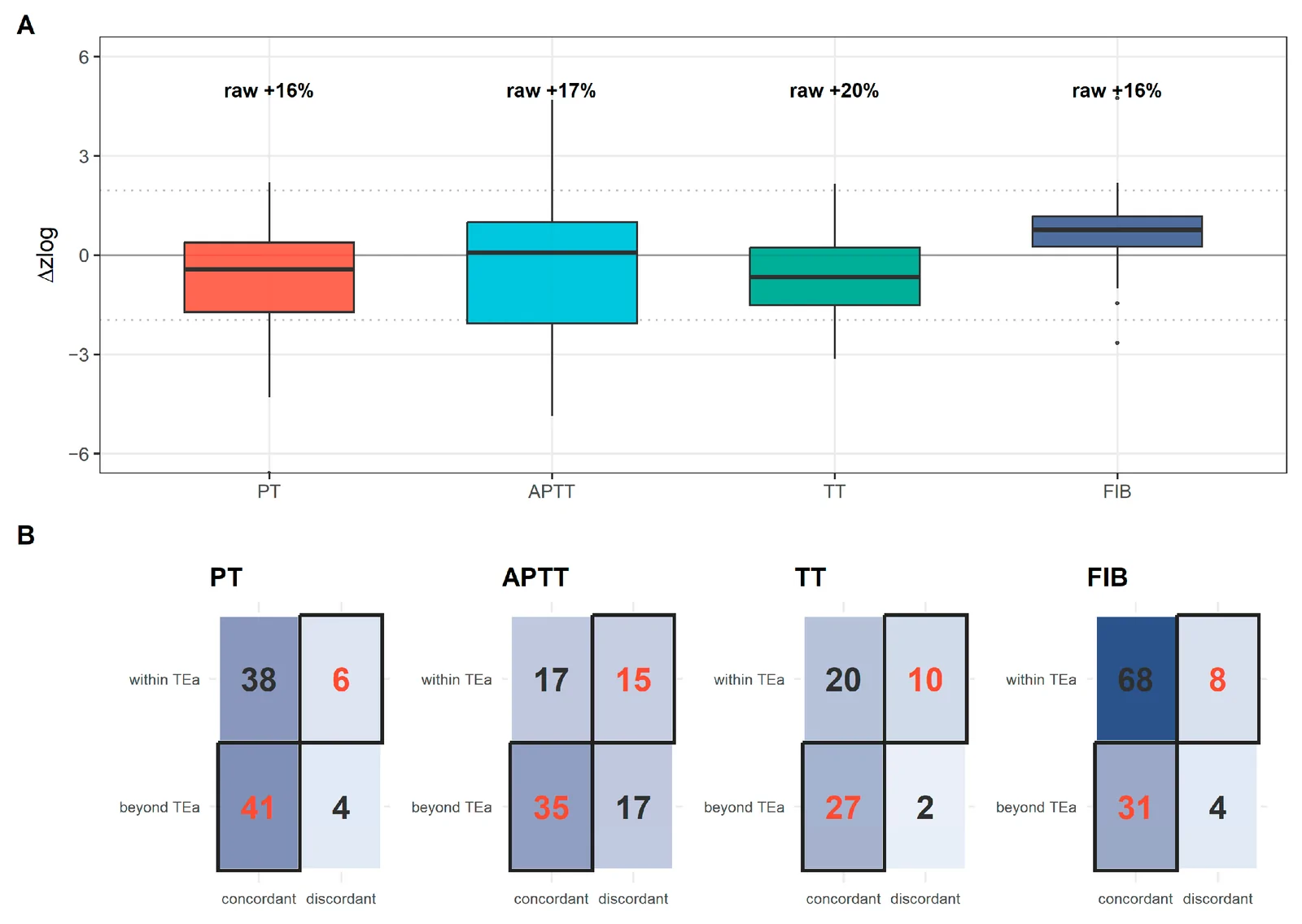
Standardized RI position and analytical-by-interpretive classification. (**A**) Δ*zlog* by assay; dotted horizontal lines mark the standardized RI limits (−1.96 and +1.96), and the value above each box is the mean between-platform bias. (**B**) 2 × 2 matrices for PT, APTT, TT, and FIB, with rows indicating within/beyond TEa and columns indicating concordant/discordant RI classification. Heavy cell borders and red counts highlight the two clinically informative off-diagonal patterns: within TEa but discordant and beyond TEa but concordant. TT uses the non-enriched primary set (*n* = 59). Box colors in panel A correspond to the four assays indicated on the x-axis. In panel B, darker blue shading indicates larger cell counts, with the exact counts shown in each cell.

Interpretive divergence was summarized using concordance of reference-interval classification (Table 3) and the analytical-by-interpretive 2 × 2 matrix (Figure 3B). Continuous Δ*zlog* values were used as supportive descriptors of standardized reference-interval position, with median values of −0.43 for PT, +0.09 for APTT, −0.66 for TT, and +0.77 for FIB (Table 3; Figure 3A and Figure S1).

**Figure 4:**
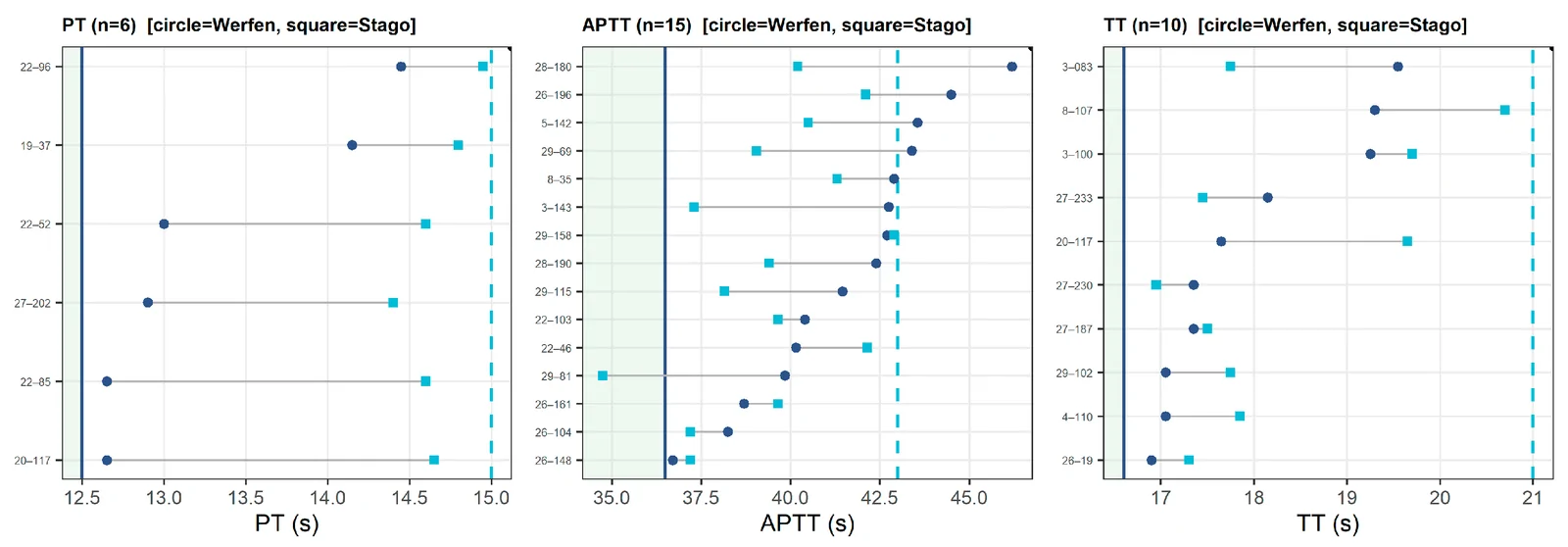
RI offset near the upper reference limit for PT, APTT, and TT. Each segment links a specimen’s paired result on Werfen (circle) and Stago (square), restricted to specimens that were within TEa but interpretively discordant. Vertical lines identify the platform-specific upper RI limits (solid, Werfen; dashed, Stago), and the shaded band marks values within the Werfen RI; sample IDs are shown. These threshold cues show how numerically close results can receive different flags. TT uses the non-enriched primary set (*n* = 59).

Two main mechanisms of interpretive divergence were identified. The first was reference-interval offset, most evident for TT. The Stago TT reference interval was higher than the Werfen interval; therefore, specimens with TT values between 16.6 and 21.0 s were above the Werfen upper reference limit but within the Stago reference interval, leading to discordant reference-interval classifications (Figure 4) and an interpretive discordance of 20.3%. Because this offset was concentrated near the upper reference limit rather than reflected as a uniform displacement of the entire distribution, its effect was more apparent among the discordantly classified specimens shown in Figure 4 than from the median Δ*zlog* alone. A negative Δ*zlog* indicates that the same specimen occupied a lower standardized position relative to the Stago reference interval than to the Werfen reference interval.

The second mechanism was analytical dispersion, most evident for APTT. Although the median Δ*zlog* for APTT was close to zero (+0.09), the distribution was wide, and interpretive discordance reached 38.1%, indicating that discordance was mainly associated with between-platform scatter rather than a systematic reference-interval offset. FIB showed a different pattern: because both platforms used the same reference interval for FIB, discordance and Δ*zlog* displacement were mainly attributable to proportional bias crossing the shared upper reference limit. INR and AT, included as secondary comparator assays, showed small Δ*zlog* values and high interpretive concordance, consistent with their closer numerical agreement and near-identical reference intervals.

The analytical-by-interpretive 2 × 2 matrix (Figure 3B) contained observations in both clinically informative cells for each clotting-time assay: specimens that exceeded TEa but retained concordant reference-interval classifications, and specimens that remained within TEa but showed discordant reference-interval classifications. The former indicates that an analytical difference beyond the allowable error did not necessarily alter interpretation under analyzer-specific reference intervals, whereas the latter indicates that numerically similar paired results could still fall on opposite sides of the decision limits of the two platforms. This within-TEa but interpretively discordant pattern was most evident for APTT and TT (Figure 4). For TT, interpretive discordance was 20.3% in the non-enriched primary sample (*n* = 59) and increased to 42.7% in the Werfen-prolonged enrichment set (*n* = 89; Figure S4), consistent with the expected effect of one-platform enrichment and not interpretable as clinical prevalence. The interpretive classification, represented by the matrix columns, was determined by analyzer-specific reference intervals and was therefore independent of the TEa threshold, whereas TEa determined only the analytical row classification.

In summary, these findings demonstrate that numerical agreement and RI-based interpretation were not interchangeable. TT most clearly demonstrated RI-offset-driven discordance, whereas APTT showed broad between-platform dispersion around a near-zero median Δ*zlog*; the matrix also identified specimens for which TEa status and interpretive status pointed in different directions.

## 4. Discussion

### 4.1. Mechanisms of Numerical and Interpretive Divergence

Numerical comparability and reference-interval-based interpretation were related but distinct dimensions of analyzer comparability. Similar magnitudes of numerical bias, approximately +16% for several primary assays, were associated with different interpretive consequences: interpretive concordance ranged from 61.9% for APTT to 89.2% for FIB, and the analytical-by-interpretive 2 × 2 matrix contained observations in both clinically informative cells. Specifically, some specimens exceeded TEa but retained concordant reference-interval classifications, whereas others remained within TEa but were classified differently by the two platforms.

Two main mechanisms accounted for this divergence. The first was reference-interval offset, most evident for TT. The Stago TT reference interval was higher than the Werfen interval, creating a 16.6–21.0 s zone in which results were above the Werfen upper reference limit but within the Stago reference interval (Figure 4). This interval configuration produced discordant reference-interval classifications despite numerically similar paired results. The second mechanism was analytical dispersion, most evident for APTT. In this assay, the median Δ*zlog* was close to zero, but the distribution was wide, indicating that discordance was mainly due to between-platform scatter rather than a systematic reference-interval offset. FIB showed a complementary pattern: because the two platforms used the same reference interval for FIB, interpretive divergence was mainly attributable to proportional bias crossing the shared decision limit.

The contribution of the *zlog* transformation in this study is not the replacement of raw reference-limit classification with *zlog* classification, because classification at *zlog* thresholds of ±1.96 is equivalent to classification using the original reference limits. Rather, its value lies in providing a continuous descriptor of standardized reference-interval position that can be interpreted alongside the analytical-by-interpretive matrix. Because the *zlog* scaling factor is inversely related to reference-interval width, Δ*zlog* magnitudes should not be compared directly across assays. Accordingly, Δ*zlog* was interpreted primarily by its direction and distribution, rather than by ranking assays according to absolute Δ*zlog* values.

APTT showed the lowest interpretive concordance, consistent with wide analytical dispersion around a near-zero median Δ*zlog.* This finding extends previous reports that optical and mechanical coagulation methods may correlate well but are not necessarily interchangeable [1,2,3], by showing how such non-interchangeability can translate into patient-level differences in reference-interval classification [6,7]. Documented anticoagulant or antiplatelet therapy was present in only three specimens, and sensitivity analyses excluding the warfarin and rivaroxaban specimens did not materially change the PT or APTT findings. Therefore, documented anticoagulant exposure does not appear to account for the observed divergence, although this mechanism warrants dedicated evaluation in anticoagulated populations, particularly because direct oral anticoagulants can have reagent-dependent effects on clotting assays [15].

The secondary comparator assays provided additional context. Although PT in seconds showed a between-platform bias of +15.6%, INR showed close numerical agreement, with a bias of −1.1%. The two PT reagent systems differed in thromboplastin source and calibration: STA-Neoplastine CI Plus on Stago uses rabbit-brain thromboplastin with ISI 1.36, whereas HemosIL RecombiPlasTin 2G on Werfen uses recombinant human tissue factor with ISI 1.01. Because INR applies reagent-specific ISI calibration to PT, the near-zero INR bias provides an internal demonstration that much of the difference in PT seconds reflects reagent sensitivity and calibration rather than a uniform analyzer-wide shift. This standardization improves comparability for INR, but it does not make raw PT seconds interchangeable across platforms. Accordingly, laboratories using multiple platforms should interpret longitudinal changes in raw PT seconds in the context of the reagent–ISI system, even when INR results appear comparable. Thus, the raw PT result in seconds differed between platforms, whereas the ISI-based INR calculation substantially reduced the platform difference. This finding emphasizes that comparability should be assessed for the clinically interpreted quantity, not only for the underlying raw measurement. Similarly, the APTT divergence cannot be explained simply by different activator classes, because both platforms used silica-based APTT reagents; the observed differences are therefore more likely related to reagent formulation, detection principle, and platform-specific analytical behavior rather than activator class alone.

Our findings are consistent with the independent Stago-versus-Werfen comparison reported by Denitto et al. [3], who compared the mechanical sthemO 301 with the optical ACL TOP 750. They also observed higher PT results on the mechanical analyzer (+13.7% for PT in seconds, compared with +15.6% in the present study), significant between-analyzer bias for routine coagulation assays except antithrombin, and the weakest agreement for APTT. The consistency of these observations across a different mechanical Stago platform suggests that the direction and approximate magnitude of optical-versus-mechanical differences may not be specific to the particular instrument pair evaluated in this study.

### 4.2. Clinical Implications and Laboratory Implementation

Concrete scenarios illustrate the potential consequence of discordance. A TT result of 18 s is above the Werfen upper RI limit of 16.6 s but remains within the Stago RI of 14–21 s; if analyzer identity is ignored, the same numerical result could trigger further evaluation on one platform but be reported without a high flag on the other. Likewise, for serial APTT values near an upper RI limit, a change in platform could make an analytical difference appear to be a biological change—or conceal a true change—thereby complicating decisions about repeat testing or additional investigation. These examples illustrate classification risk and are not intended as direct patient-outcome estimates.

For laboratory practice, these findings support a two-step approach to platform interchangeability and within-laboratory harmonization [26]. First, numerical bias should be characterized across the relevant analytical range using method-comparison procedures, such as Passing–Bablok regression and Bland–Altman analysis, with bias evaluated relative to TEa. Second, the interpretive consequence of between-platform differences should be assessed using verified analyzer-specific reference intervals, including the proportion of specimens with discordant reference-interval classifications near clinical decision limits. A platform pair may appear acceptable numerically but still produce clinically relevant interpretive discordance, or conversely may exceed TEa without changing reference-interval classification. When interpretive discordance is concentrated near a decision limit, as observed for TT in this study, laboratories may consider adopting a common locally established reference interval, restricting the affected assay to a single platform for longitudinal monitoring, or adding interpretive comments for platform-dependent results near the decision limit. The appropriate strategy depends on the strength of local reference-interval verification, the assay’s purpose, and its clinical use.

For routine quality assurance, laboratories can implement the framework as a periodic, assay-specific workflow: (1) select paired specimens spanning the RI and relevant decision limits; (2) characterize numerical behavior with Passing–Bablok and Bland–Altman analyses; (3) cross-classify specimen-level TEa status and RI-based concordance in the analytical-by-interpretive matrix; and (4) inspect the direction and spread of Δ*zlog* to distinguish systematic RI offset from heterogeneous analytical dispersion. Persistent offset should prompt review of RI harmonization or platform-specific comments, whereas broad dispersion should prompt investigation of reagents, calibration, preanalytical factors, and whether serial monitoring should remain on one platform.

### 4.3. Limitations and Future Directions

This study has several limitations. First, it was a single-center study using a purposive, non-probability sample selected to cover the measurement and interpretive range rather than the routine distribution; the observed discordance rates are therefore sample-specific and not prevalence estimates. The TT enrichment set was conditional on Werfen prolongation and was used only for sensitivity and mechanistic illustration. Second, the RIs were manufacturer-specified and verified locally by small-sample transference rather than established de novo. Individual-level demographic summaries and exact out-of-range counts from the verification cohorts were not retained with the paired dataset, limiting retrospective description of the reference population. Third, undocumented anticoagulant exposure could not be excluded, and harmonized interference-index data for hemolysis, icterus, and lipemia were unavailable; these medication and sample-quality factors could have contributed to individual between-platform differences. Fourth, Passing–Bablok regression was unstable for APTT and TT because of limited effective range, clustered values or ties, and substantial scatter. Fifth, only two analyzer–reagent systems were evaluated. Finally, *zlog* classification at ±1.96 is equivalent to classification at the original RI limits; the added value lies in combining continuous Δ*zlog* with the analytical-by-interpretive matrix, not in creating a new classifier. Future prospective studies should include multiple centers, routine consecutive samples, dedicated anticoagulated cohorts, and additional analyzer–reagent pairs, and should test whether harmonized RIs, single-platform longitudinal testing, or platform-specific interpretive comments reduce clinically relevant discordance without compromising sensitivity.

## 5. Conclusions

Numerical comparability and RI-based interpretation represent distinct, although related, dimensions of coagulation analyzer comparability. In this study, an analytical-by-interpretive 2 × 2 matrix, supported by continuous Δ*zlog* analysis, identified interpretive divergence not captured by bias-versus-TEa assessment alone. The observed mechanisms included RI offset, illustrated by TT, and analytical dispersion, illustrated by APTT. Laboratories using multiple platforms should therefore combine numerical method comparison with assessment of RI-based classification and adopt assay-specific actions—such as RI review, single-platform serial testing, or interpretive comments—when systematic discordance is identified.

## Figures and Tables

**Table 1:** Reference intervals and allowable total error.

Analyte	Werfen RI	Stago RI	TEa (Lab)	BV-Desirable TEa *
PT (s)	9.4–12.5	11–15	±15%	~4%
APTT (s)	25.1–36.5	28.5–43	±15%	~7%
TT (s)	10.3–16.6	14–21	±20%	n/a
FIB (g/L)	2.0–4.0	2.0–4.0	±20%	~14%
INR	0.8–1.2	0.8–1.2	±20%	n/a
AT (%)	83–128	80–120	±20%	n/a

Reference intervals were manufacturer-specified and locally verified by transference according to CLSI EP28-A3C and WS/T 402-2024; none required de novo establishment. TEa values were based on the Chinese national external quality assessment scheme of the National Center for Clinical Laboratories. * BV-desirable TEa values are biological-variation-derived desirable analytical-performance specifications shown for context only; the locally adopted NCCL TEa limits were used as the primary analytical acceptance criteria. RI, reference interval; TEa, total allowable error; BV, biological variation. n/a, not available.

**Table 2:** Numerical comparability (Stago vs. Werfen).

Analyte	*N*	PB Slope (95% CI)	PB Intercept	BA Bias %	BA 95% LoA	Predicted Decision-Point Bias (%)	Within TEa % (95% CI)
PT	89	0.817 (0.768, 0.860)	+4.42	+15.6%	−6% to +37%	+17.0%	49 (39, 60)
APTT	84	0.989 (0.702, 1.348) †	+5.93	+16.6%	−33% to +67%	+15.1%	38 (28, 49)
TT	59	0.823 (0.586, 1.180) †	+5.58	+20.4%	−12% to +53%	+15.9%	51 (38, 63)
FIB	111	1.426 (1.359, 1.495)	−0.95	+15.8%	−20% to +52%	+18.8%	68 (59, 76)
INR	89	0.941 (0.880, 1.000)	+0.05	−1.1%	−15% to +13%	−1.9%	98 (92, 99)
AT	54	0.786 (0.725, 0.847)	+19.77	+1.6%	−16% to +20%	−5.0%	96 (87, 99)

TT primary *n* = 59. Decision point = upper reference limit. TEa: ±15% (PT, APTT), ±20% (TT, FIB, INR, AT). † Passing–Bablok slope for APTT and TT is poorly identified (limited measurement range or high scatter) and must not be read as agreement; rely on the Bland–Altman bias.

**Table 3:** Interpretive concordance and standardized reference-interval position (Δ*zlog*, own-RI).

Analyte	*N*	Interpretive Concordance % (95% CI)	Interpretive Discordance % (95% CI)	Kappa (95% CI)	Median Δ*zlog* (Bootstrap 95% CI)	2 × 2 inC/inD/outC/outD
PT	89	88.8 (81, 94)	11.2 (6, 19)	0.78 (0.64, 0.89)	−0.43 (−0.88, −0.03)	38/6/41/4
APTT	84	61.9 (51, 72)	38.1 (28, 49)	0.24 (0.03, 0.45)	+0.09 (−0.97, +0.45)	17/15/35/17
TT	59	79.7 (68, 88)	20.3 (12, 32)	0.30 (0.00, 0.58)	−0.66 (−0.88, −0.18)	20/10/27/2
FIB	111	89.2 (82, 94)	10.8 (6, 18)	0.88 (0.81, 0.94)	+0.77 (+0.59, +0.93)	68/8/31/4
INR	89	92.1 (85, 96)	7.9 (4, 15)	0.84 (0.71, 0.93)	−0.15 (−0.33, +0.05)	80/7/2/0
AT	54	92.6 (82, 97)	7.4 (3, 18)	0.85 (0.71, 0.96)	+0.51 (+0.29, +0.73)	49/3/1/1

TT primary *n* = 59; in the enrichment set (*n* = 89), TT discordance was 42.7%. Kappa is secondary because the sampled abnormal-result distribution is not representative. In the final column, values are ordered as inC/inD/outC/outD: inC = within TEa and interpretively concordant; inD = within TEa but interpretively discordant; outC = beyond TEa but interpretively concordant; and outD = beyond TEa and interpretively discordant.

## Data Availability

The datasets generated and analyzed during the current study are available from the corresponding author on reasonable request.

## Supplementary Materials

The following supporting information can be downloaded at https://www.mdpi.com/article/10.3390/diagnostics16162511/s1, Figure S1: Analyzer-specific *zlog* values for PT, APTT, TT and FIB. Each point is a specimen plotted by its Werfen (x) and Stago (y) *zlog* value; diagonal, line of identity; dashed lines, reference limits (±1.96); red points, interpretively discordant pairs. TT, non-enriched set (*n* = 59); Figure S2: Passing–Bablok residuals versus the Werfen measurement for PT, APTT, TT, and FIB. Horizontal line, zero residual. TT, non-enriched set (*n* = 59).; Figure S3: Exploratory D-dimer comparison (*n* = 25). Log-ratio Bland–Altman plot (Stago vs. Werfen): mean bias (solid), 95% limits of agreement (dashed); Figure S4: TT sensitivity analysis. Analytical-by-interpretive 2 × 2 matrices for (A) the non-enriched primary set (*n* = 59) and (B) the Werfen-prolonged enriched set (*n* = 89); bordered cells with red counts as in Figure 3B.

## Glossary

**Term**	**Definition**
Analytical-by-interpretive matrix	A 2 × 2 framework that cross-classifies each specimen according to whether the absolute between-platform difference is within or beyond TEa and whether the two platforms yield concordant or discordant RI-based classifications.
Δ*zlog*	The difference in standardized reference-interval position between analyzers, calculated as the Stago *zlog* minus the Werfen *zlog*. A positive value indicates a higher standardized position on Stago; a negative value indicates a lower standardized position on Stago.
Interpretive concordance	Agreement between the two platforms in assigning the same category relative to each analyzer’s own RI (for example, below, within, or above the interval, as applicable).
RI	Reference interval; the lower-to-upper range of expected values for a defined reference population, verified for each analyzer-reagent system and used here for platform-specific result classification.
TEa	Allowable total error; the prespecified threshold used to classify an absolute between-platform difference as analytically acceptable (within TEa) or unacceptable (beyond TEa).
*zlog*	A logarithmically transformed, dimensionless measure of a result’s position within an analyzer-specific RI; the lower and upper reference limits are mapped to −1.96 and +1.96, respectively.
