# Supplementary material for: Numerical and Interpretive Comparability of Optical and Mechanical Coagulation Analyzers: A Zlog-Supported Method Comparison Study
Source: Diagnostics (Basel). 2026 Aug 9;16(16):2511. doi: 10.3390/diagnostics16162511 (PMC13511516; doi:10.3390/diagnostics16162511)
Supplement: Supplementary file 1 [file diagnostics-16-02511-s001.zip › Supplementary_Materials.pdf]

**Supplementary Materials**

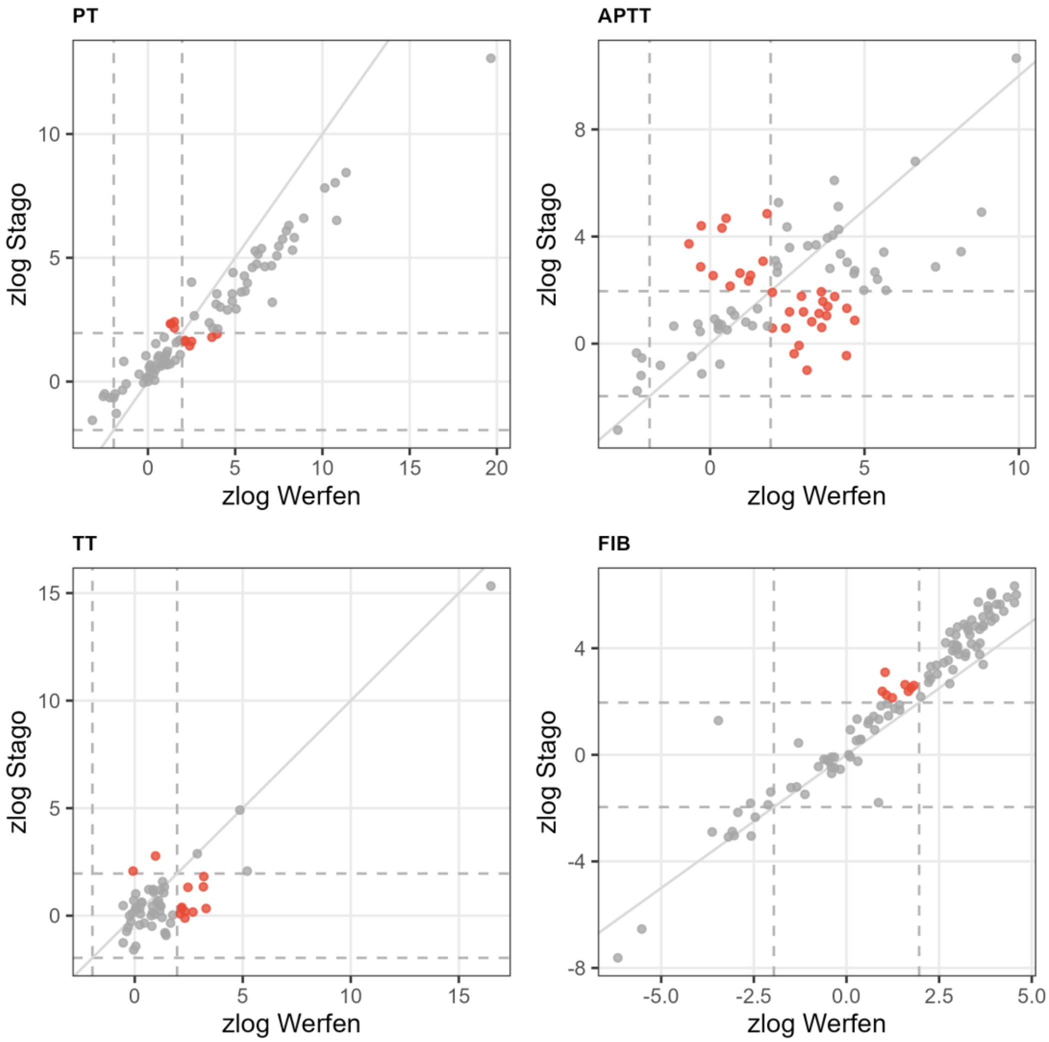


**Figure S1. Analyzer-specific zlog values for PT, APTT, TT and FIB.** Each point is a specimen plotted by its Werfen (x) and Stago (y) zlog value; diagonal, line of identity; dashed lines, reference limits (±1.96); red points, interpretively discordant pairs. TT, non-enriched set ($n$=59).

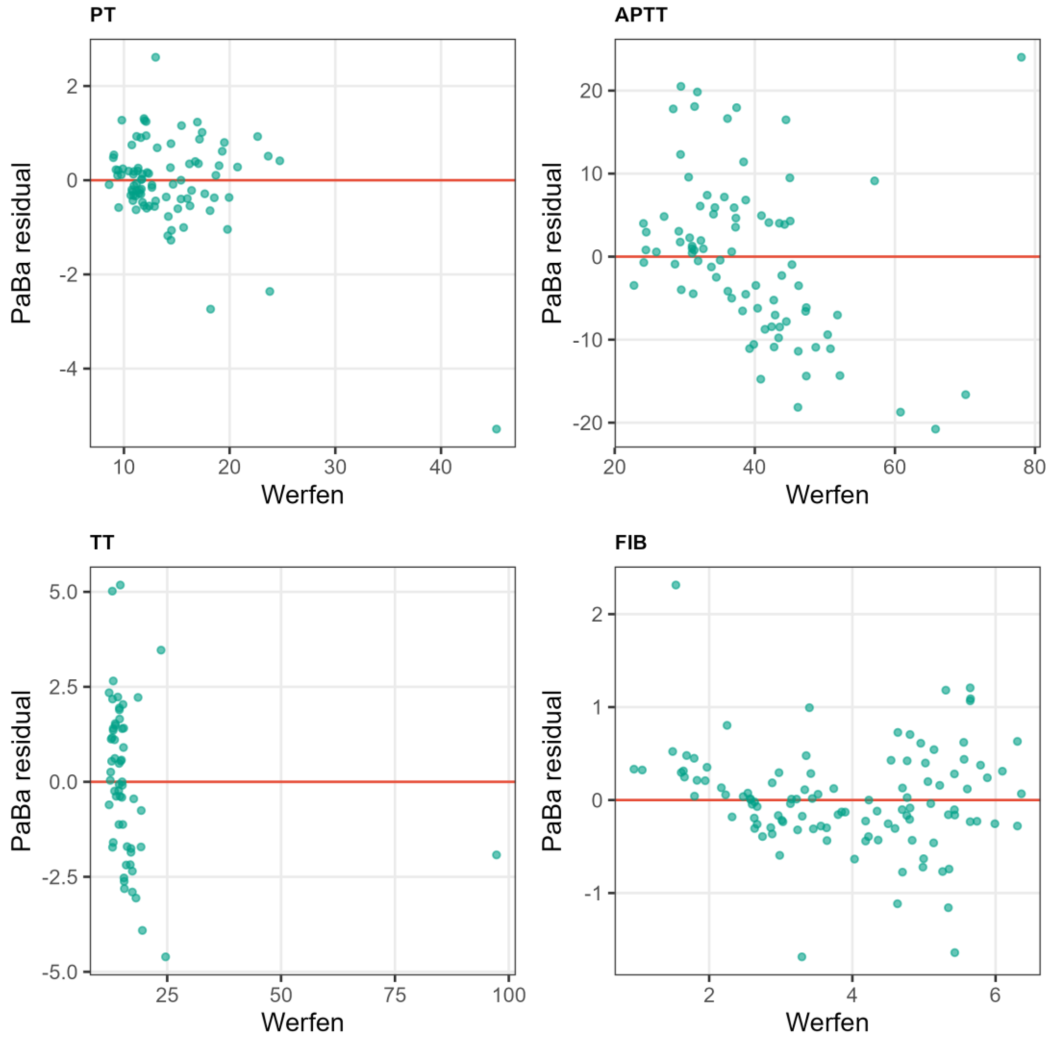


**Figure S2. Passing–Bablok residuals versus the Werfen measurement for PT, APTT, TT and FIB.** Horizontal line, zero residual. TT, non-enriched set (*n*=59).

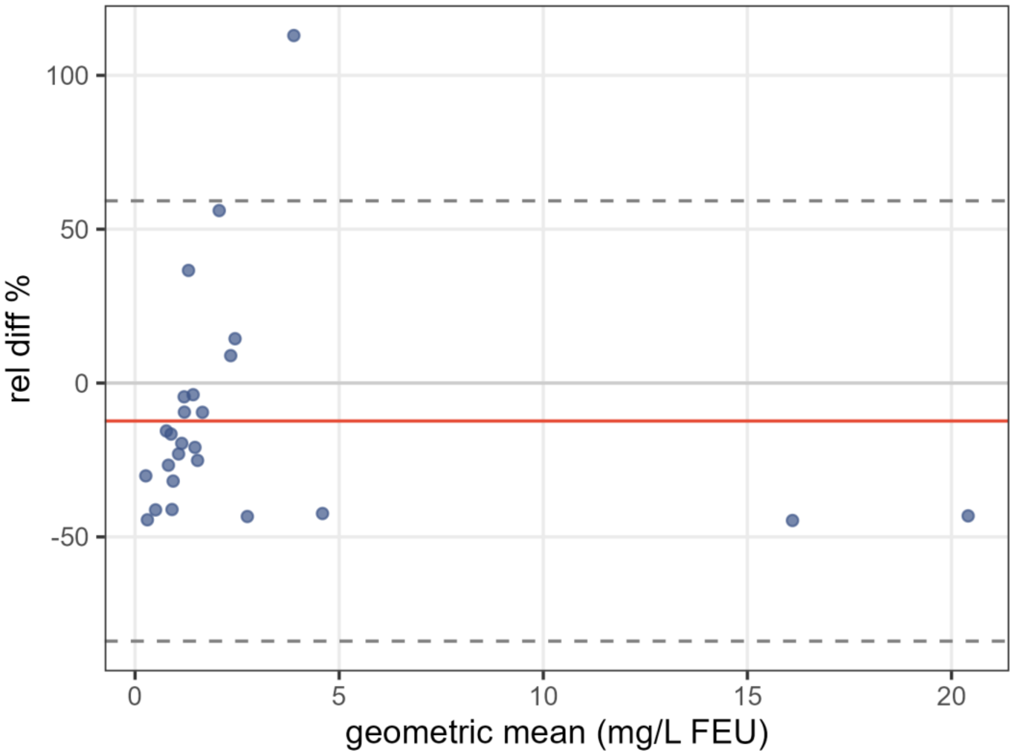


**Figure S3. Exploratory D-dimer comparison (*n*=25).** Log-ratio Bland–Altman plot (Stago vs Werfen): mean bias (solid), 95% limits of agreement (dashed).

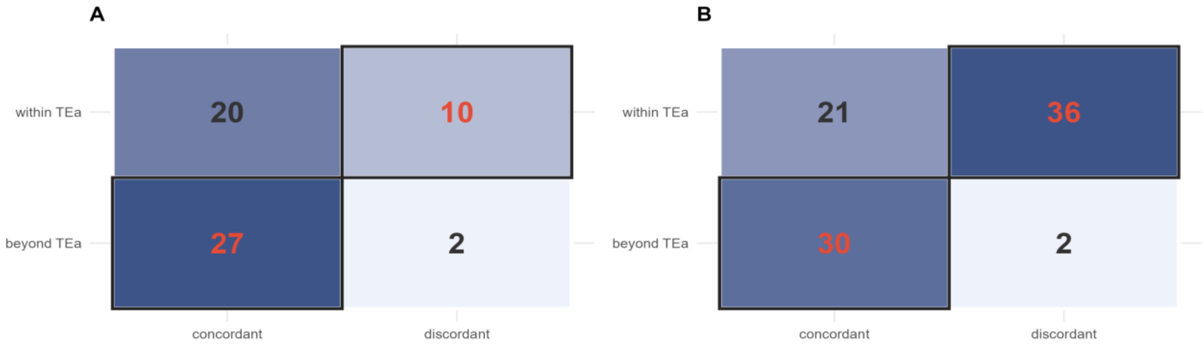


**Figure S4. TT sensitivity analysis.** Analytical-by-interpretive 2×2 matrices for (A) the non-enriched primary set (n=59) and (B) the Werfen-prolonged enriched set (*n*=89); bordered cells with red counts as in Figure 3B.
